# Supplementary material for: Construction of VAE-GRU-XGBoost intrusion detection model for network security
Source: PLoS One. 2025 Jun 25;20(6):e0326205. doi: 10.1371/journal.pone.0326205 (PMC12193923; doi:10.1371/journal.pone.0326205)
Supplement: S1 File — (DOC) [file pone.0326205.s001.doc]

**The data in Figure 7**

| Epochs | Refactoring loss | | |
| --- | --- | --- | --- |
|  | GRU Improved VAE Model | VAE model before GRU improvement |  |
| 0 | 0.1088 | 0.1135 |  |
| 20 | 0.0431 | 0.0718 |  |
| 40 | 0.0308 | 0.0711 |  |
| 60 | 0.0195 | 0.0443 |  |
| 80 | 0.0175 | 0.0468 |  |
| 100 | 0.0174 | 0.0410 |  |

**The data in Figure 8**

| Epochs | Extraction accuracy/% | | | | Extraction accuracy/% | | | | Extraction accuracy/% | | | |
| --- | --- | --- | --- | --- | --- | --- | --- | --- | --- | --- | --- | --- |
|  | GRU Improved VAE | XGBoost | SVM | GAN | GRU Improved VAE | XGBoost | SVM | GAN | GRU Improved VAE | XGBoost | SVM | GAN |
| 0 | 0 | 0 | 0 | 0 | 0 | 0 | 0 | 0 | 0 | 0 | 0 | 0 |
| 20 | 87.65 | 83.28 | 74.55 | 67.50 | 95.04 | 85.95 | 60.72 | 61.37 | 94.27 | 87.05 | 72.67 | 68.03 |
| 40 | 90.99 | 84.95 | 77.80 | 74.28 | 95.00 | 89.62 | 68.10 | 64.11 | 95.15 | 87.18 | 75.67 | 69.09 |
| 60 | 92.94 | 86.44 | 76.13 | 70.28 | 92.83 | 88.94 | 72.24 | 68.72 | 94.54 | 87.59 | 77.66 | 73.49 |
| 80 | 92.85 | 88.20 | 77.80 | 74.74 | 95.05 | 89.09 | 76.38 | 69.88 | 94.30 | 87.35 | 77.60 | 73.62 |
| 100 | 94.19 | 88.63 | 79.54 | 77.25 | 95.07 | 89.22 | 78.17 | 76.20 | 94.28 | 88.06 | 77.64 | 75.83 |

**The data in Figure 9**

| Traffic subclass | Detection rate/% | | | |
| --- | --- | --- | --- | --- |
|  | GRU Improved VAE | XGBoost | SVM | GAN |
| Normal | 96.83 | 95.29 | 92.04 | 92.63 |
| DoS | 87.24 | 81.47 | 58.28 | 74.25 |
| Probe | 81.81 | 58.70 | 42.11 | 50.93 |
| R2L | 50.96 | 37.86 | 9.89 | 13.57 |
| U2R | 42.87 | 37.91 | 5.50 | 8.24 |

**The data in Figure 11**

| False Positive Rate/% | KDD99 dataset True Positive Rate/% | | | | OODS dataset True Positive Rate/% | | | |
| --- | --- | --- | --- | --- | --- | --- | --- | --- |
|  | GRU Improved VAE | XGBoost | SVM | GAN | GRU Improved VAE | XGBoost | SVM | GAN |
| 0 | 0 | 0 | 0 | 0 | 0 | 0 | 0 | 0 |
| 10 | 91.33 | 74.56 | 73.80 | 62.48 | 83.96 | 75.58 | 58.81 | 29.58 |
| 20 | 92.57 | 82.05 | 79.39 | 71.12 | 93.08 | 85.31 | 72.12 | 45.56 |
| 30 | 94.23 | 83.41 | 80.63 | 74.15 | 96.10 | 89.74 | 82.88 | 53.80 |
| 40 | 94.45 | 84.31 | 81.75 | 75.93 | 99.24 | 92.38 | 85.79 | 65.82 |
| 50 | 94.58 | 86.05 | 87.08 | 77.93 | 99.71 | 95.90 | 89.57 | 75.09 |
| 60 | 94.80 | 92.12 | 88.09 | 83.25 | 100 | 96.63 | 89.93 | 81.01 |
| 70 | 94.78 | 92.88 | 90.09 | 85.02 | 100 | 97.37 | 89.75 | 87.23 |
| 80 | 95.03 | 95.03 | 90.08 | 87.79 | 100 | 98.26 | 90.86 | 89.34 |
| 90 | 95.51 | 95.67 | 90.94 | 91.32 | 100 | 98.99 | 93.51 | 96.17 |
| 100 | 95.88 | 95.76 | 92.45 | 92.83 | 100 | 100 | 100 | 100 |
