# Supplementary material for: Construction of VAE-GRU-XGBoost intrusion detection model for network security
Source: PLoS One. 2025 Jun 25;20(6):e0326205. doi: 10.1371/journal.pone.0326205 (PMC12193923; doi:10.1371/journal.pone.0326205)
Supplement: Attachment — Submitted filename: Response to comments.docx [file pone.0326205.s002.docx]

Review Comments to the Author

Please use the space provided to explain your answers to the questions above. You may also include additional comments for the author, including concerns about dual publication, research ethics, or publication ethics. (Please upload your review as an attachment if it exceeds 20,000 characters)

Reviewer #1:

Overview

This manuscript proposes a novel hybrid model for network intrusion detection that combines VAE, GRU, and XGBoost. Their model shows great performance at feature extraction and classification of network traffic to detect intrusions on the KDD99 and OODS datasets, as well as efficient processing times. The authors also include detailed literature review, discussions and ablation study.

Major Issues

1. Insufficient Technical Details: The paper lacks a precise mathematical explanation of how VAE, GRU, and XGBoost components are integrated. While individual components are explained, their interconnection isn't clearly articulated. Implementation details such as hyperparameters, training strategies, and optimization techniques are missing for the proposed methods, as well as compared methods like SVM, GAN and CNN.

Also, some of the math equations seem vague. For example, Equation (1) and (2) seem to imply that both the encoder and the decoder contain just one hidden layer respectively. There is also no clear label of the shape of input, parameters and output.

The feature selection accuracy mentioned around Figure 8 is not clearly defined.

Reply: Thank you for your professional comments. The main revisions are: (1) The precise mathematical explanation of how VAE, GRU, and XGBoost components are integrated in the method, as well as their interrelationships, have been supplemented. (2) Implementation details such as hyperparameters, training strategies, and optimization techniques have been added to the manuscript. (3) Equations (1) and (2) have been carefully verified, and in order to avoid ambiguity, the equations have been modified and detailed explanations have been added. (4) The accuracy of feature selection mentioned in Figure 8 has been clearly defined in the manuscript. The modified content is as follows:

(1) In network intrusion detection, VAE, GRU, and XGBoost are integrated together to form a powerful multi-component model. Firstly, VAE is used for unsupervised feature learning, which maps input data to latent space through an encoder to learn a low dimensional representation of the data. In order to further enhance the model's ability to capture temporal data, GRU is introduced and combined with VAE. The role of GRU is to extract time-dependent features from the input temporal data. Specifically, the input data of the encoder is first processed by a GRU network, which extracts temporal features through GRU's gating mechanism. These temporal features are then fed into VAE for latent spatial mapping, forming a more refined feature representation. Finally, XGBoost serves as a classifier to receive feature representations processed by VAE and GRU, and complete the final network intrusion classification task. XGBoost achieves efficient intrusion detection by learning and classifying these extracted features. In summary, VAE, GRU, and XGBoost work together in this model. VAE is responsible for unsupervised feature learning, GRU enhances temporal feature extraction, and XGBoost completes intrusion classification, forming a collaborative intrusion detection system.

(2) The key hyperparameters in the VAE-GRU-XGBoost based network intrusion detection model are set as follows: the learning rate of the VAE module is 0.001, the batch size is 64, and the latent dimension is 50; For GRU, the hidden layer dimension is set to 128, the learning rate is set to 0.001, and the Adam optimizer is used for training; For XGBoost, use the default learning rate and tree depth of 6. During the training process, cross validation is used to optimize these hyperparameters to ensure the best performance of the model. In order to improve training efficiency, batch training and early stopping strategies were adopted. During the training process, if the loss function of the validation set does not improve within a certain number of rounds, the training will be stopped.

(3) The encoder consists of multiple hidden layers, with the goal of mapping the input to latent space. The output of the encoder is the latent variable $z$, which represents the distribution of input data in the latent space. The mathematical formula of VAE encoder is shown in equation (1)

 (1)

In equation (1), $u\left(x\right)$ and $\sigma^{2}\left(x\right)$ represent the mean and standard deviation, respectively. The encoder maps the input $x$ to latent space through a series of fully connected layers. The decoder maps the representation $z$ of the latent space back to the original data space and reconstructs the data. The decoder is also composed of multiple fully connected layers, and its output is the mean of the reconstructed data [20]. The mathematical expression for the decoding process is shown in equation (2).

 (2)

In equation (2), represents the reconstructed data generated by the decoder.

(4) Research calculates the classification accuracy of each feature through cross validation, and then sorts the features based on this accuracy to select the features with higher contribution. Compare the selected feature set with all features and calculate the accuracy change to measure the effectiveness of feature selection.

2. Limited Novelty and Design Justification: The authors claim novelty in combining these three techniques, but don't sufficiently explain why this particular combination was chosen over alternatives. Also in the related work section, the authors mentioned that “Cao B et al. combined deep learning technology and GRU design to improve the intrusion detection accuracy.” It’s straightforward to see the close relationship between Cao B et al.’s model and the proposed one. However, this is no discussion regarding this matter.

Also, Cao B et al.’s model can be trained end-to-end as they used a softmax layer after all feature extraction steps, while the authors use XGBoost which I don’t think can be applied end to end. I would like to see more clarification/discussion on this choice.

The feature selection process discussed around Figure 4 needs to be justified as well. The described process could be highly dependent on the order of added features. It would be good to see if different order gives similar performance.

Reply: Thank you for your professional comments. The reason for choose the specific combination has been explained in the manuscript. The differences between Cao B et al.'s model and the method proposed in this paper have been discussed in detail in the relevant work section. The content of the XGBoost method has been carefully verified. As an ensemble learning model, XGBoost usually does not support end-to-end training, but is trained based on preprocessed features. Therefore, the semantic explanation should be unclear, and clearer explanations have been added to the manuscript. The design concept of the feature selection process mentioned in Figure 4 is based on XGBoost's feature importance ranking to select features, which ensures that each added feature contributes the most to the model performance. A more detailed explanation has been added to the manuscript regarding the feature selection process. Please see the revisions as follows:

(1) In order to improve the accuracy of network intrusion detection, three technologies, GRU, VAE, and XGBoost, were studied and combined. In the model design, GRU was used to capture temporal features, VAE was used for latent feature extraction, and XGBoost was used as a classifier for accurate classification. The reason for choosing this specific combination is that GRU can effectively capture time series features in the input data, avoiding the gradient vanishing problem in traditional RNNs. GRU has lower computational complexity compared to traditional LSTM and RNN when dealing with long-term dependencies, thus improving the real-time performance of network intrusion detection. VAE can extract potential feature representations from data through variational autoencoders, especially on high-dimensional data, where VAE has strong feature representation capabilities. As an ensemble learning algorithm based on decision trees, XGBoost can perform accurate classification on existing features, especially suitable for classification tasks of high-dimensional data.

(2) For example, Cao B et al.'s model extracts spatial features through convolutional neural networks (CNN) and then uses softmax for classification. Although there are some similarities between this study and the model presented in this article, there are differences in their methods. The model in this article uses GRU to capture temporal features and adopts XGBoost classifier, emphasizing stronger classification ability in complex temporal data and high-dimensional features.

(3) The GRU and VAE parts are responsible for extracting and encoding data features, while XGBoost serves as a classifier to optimize the extracted features. Although end-to-end training is not possible, this design can classify through effective ensemble methods after feature extraction, which can improve the accuracy and stability of the model.

(4) The importance of features is determined by calculating the contribution of each feature in the decision tree. Features with higher importance usually provide more valuable information, thus having a greater impact on the classification ability of the model. In order to reduce computational complexity and improve classifier performance, the study adopted a method of adding features in order of their importance. Since the importance of features is calculated based on their contribution to predicting the target variable during model training, theoretically, the more important features have a greater impact on performance improvement. Therefore, the study did not conduct experiments on all possible feature addition orders, but instead determined the order of feature addition based on the calculated feature importance ranking to optimize the efficiency and performance of the model. The feature sequence experiments have been added as follows:

In order to verify the impact of different feature addition orders on model performance, a series of experiments were conducted by randomly arranging input features, and the feature addition order and corresponding AUC values of each experiment were recorded. The experimental results of different feature addition orders are shown in Table 7. From Table 7, it can be seen that the AUC values after modifying the order are all greater than 0.80. However, changing the order of feature addition does indeed affect the performance of the model. When adding features of lower importance first, it can lead to a decrease in AUC value. If the AUC is lower than the set threshold, it will have a negative impact on the learning performance of the model, thereby affecting the effectiveness of the results.

**Table 7. Experimental results of different feature addition orders**

| Experiment number | Feature addition order | AUC Value | Above Threshold | Result Validity |
| --- | --- | --- | --- | --- |
| 1 | src_bytes → protocol_type → hot → num_failed_logins → diff_srv_rate | 0.85 | Yes | Valid |
| 2 | protocol_type → hot → num_failed_logins → diff_srv_rate → src_bytes | 0.83 | No | Invalid |
| 3 | hot → src_bytes → num_failed_logins → diff_srv_rate → protocol_type | 0.84 | Yes | Valid |
| 4 | diff_srv_rate → num_failed_logins → src_bytes → protocol_type → hot | 0.86 | Yes | Valid |
| 5 | num_failed_logins → diff_srv_rate → src_bytes → protocol_type → hot | 0.82 | No | Invalid |
| 6 | flag → dst_host_same_srv_rate → srv_count → dst_host_same_src_port_rate | 0.80 | No | Invalid |
| 7 | dst_host_same_srv_rate → srv_count → dst_host_same_src_port_rate → flag | 0.81 | No | Invalid |
| 8 | srv_count → dst_host_same_src_port_rate → flag → dst_host_same_srv_rate | 0.83 | Yes | Valid |
| 9 | dst_host_same_src_port_rate → srv_count → flag → dst_host_same_srv_rate | 0.84 | Yes | Valid |
| 10 | dst_host_same_srv_rate → dst_host_rerror_rate → service → dst_bytes | 0.87 | Yes | Valid |

3. Unfair Comparisons: The compared methods like standard XGBoost, SVM and GAN are relatively simple and naïve approaches, compared to the proposed method. So even if the proposed method outperforms others, there’s not enough evidence to make the intended conclusion. At least similar hybrid models should be compared.

Reply: Thank you for your professional comments. Similar mixed models have been added for comparative experiments in the result analysis.

While the LSTM XGBoost model performs well in handling various types of attacks, the proposed model offers better adaptability and generalization capabilities. However, the model presented in this article demonstrates higher adaptability and generalization ability when dealing with complex features, thanks to its VAE feature extraction mechanism. In addition, from the perspective of inference time, the LSTM XGBoost model has a longer inference time, indicating that it is not suitable for most real-time detection needs. Therefore, it can be concluded that the research model outperforms other models in terms of accuracy, speed, and robustness in real-world scenarios.

**Table 5. Performance comparison of different network intrusion detection models**

| Model | Accuracy/% | Training time/hour | Inference time/s | Robustness |
| --- | --- | --- | --- | --- |
| VAE-GRU-XGBoost | 97.58 | 1.15 | 0.003 | High adaptability |
| DAE | 93.08 | 10.51 | 0.167 | Affected by inconsistent data |
| RF | 92.06 | 3.46 | 1.644 | Not adapted to new types of attacks |
| CNN | 91.68 | 14.67 | 0.076 | More data training is needed |
| LSTM-XGBoost | 95.32 | 2.25 | 0.052 | Good adaptability with moderate data |

4. Vague Math Equations: Some of the math equations seem vague. For example, Equation (1) and (2) seem to imply that both the encoder and the decoder contain just one hidden layer respectively. There is also no clear label of the shape of input, parameters and output.

Reply: Thank you very much for your comments. Equations (1) and (2) have been carefully verified and revised. Please see details as follows:

The encoder consists of multiple hidden layers, with the goal of mapping the input to latent space. The output of the encoder is the latent variable $z$, which represents the distribution of input data in the latent space. The mathematical formula of VAE encoder is shown in equation (1)

 (1)

In equation (1), $u\left(x\right)$ and $\sigma^{2}\left(x\right)$ represent the mean and standard deviation, respectively. The encoder maps the input $x$ to latent space through a series of fully connected layers. The decoder maps the representation $z$ of the latent space back to the original data space and reconstructs the data. The decoder is also composed of multiple fully connected layers, and its output is the mean of the reconstructed data [20]. The mathematical expression for the decoding process is shown in equation (2).

 (2)

In equation (2), represents the reconstructed data generated by the decoder.

Minor Issues

1. Inadequate Dataset Description: There are no examples of data from KDD99 and OODS datasets. It would give the readers more context if some examples can be shown at least in the SI.

Reply: Thank you for your professional opinion. The manuscript has been supplemented with data examples from the KDD999 dataset, as follows:

**Table 1. Examples of traffic types and attack types in the KDD99 dataset**

| Category | Description | Attack Types |
| --- | --- | --- |
| Normal Traffic | Regular network traffic without any abnormal behavior | Normal |
| Denial of Service (DoS) | Attacks that aim to make the target service unavailable by sending large amounts of data or exploiting protocol vulnerabilities | Apache2, back, mailbomb, smurf, named, xsnoop, etc. |
| Probe Attack | Used to collect network information or perform system scans | Nmap, ipsweep, saint, mscan, etc. |
| User to Root (U2R) | Attacks that gain unauthorized root access to a system | Ps, buffer_overflow, perl, rootkit, sqlattack, etc. |
| Remote to Local (R2L) | Remote attackers exploit weak passwords or vulnerabilities to gain access to local systems | guess_passwd, ftp_write, imap, phf, warezclient, etc. |

2. Typos: In the abstract, "The results *showed* *demonstrated*..." Table 2 and Figure 11 show “XGBoos” instead of “XGBoost.”

Reply: I am so sorry for the mistake, “XGBoos” has been changed to “XGBoost.”. The results * showed * demonstrated *..." has been changed to "The results showed...".
